# Avoiding Surgery: Successful Management of a Patient With Severe Keratoconus Using Scleral Contact Lenses

**DOI:** 10.7759/cureus.90263

**Published:** 2025-08-16

**Authors:** Ahmed Almaweri

**Affiliations:** 1 Department of Optometry, Noor Alyemen Eye and E.N.T. Consulting Center, Sana'a, YEM

**Keywords:** avoiding surgery, case report, contact lens fitting, keratoconus, non-surgical management, scleral lenses, visual rehabilitation

## Abstract

Keratoconus is a degenerative and progressive corneal disease that occurs bilaterally and asymmetrically. The cornea's thinning and protrusion are its defining features. Vision is decreased as a result of these changes. This case report demonstrates how a severe keratoconus patient was managed with scleral contact lenses, thereby effectively precluding the need for surgery. Despite the challenges of this condition, scleral lenses work well. Here, I describe the case of a 34-year-old male who came to our center with a known history of severe keratoconus. His uncorrected visual acuity was counting fingers in each eye, with minimal improvement from eyeglasses. He had routine exams, and I confirmed that he was a good candidate for wearing scleral lenses. I successfully fitted this patient with mini-scleral lenses in both eyes. The fitting was optimal, which allowed the patient to achieve a 20/30 visual acuity in each eye, emphasizing the positive impact of this non-surgical approach. This case study intends to document the success of managing severe keratoconus using scleral contact lenses.

## Introduction

Keratoconus is a degenerative and progressive corneal disease that occurs bilaterally and asymmetrically. The cornea's thinning and protrusion are its defining features; in some cases, it may also lead to corneal scarring. These changes result in vision impairments [1]. The cause of keratoconus is still not completely clear; however, it is regarded as a condition influenced by multiple factors, including biochemical, genetic, and environmental components [2]. Keratoconus does not exhibit any preference for ethnicity or gender. While it is primarily an ocular disease, it may also be related to several other ocular and systemic conditions [3].

The classification of keratoconus severity is critical for progression monitoring and management. Various systems have been developed for keratoconus staging, of which the Amsler-Krumeich system is the most commonly used. Keratoconus severity, according to the Amsler-Krumeich staging, is classified into four stages: mild, moderate, advanced, and severe [4].

The management of keratoconus significantly differs depending on how advanced the condition is. Typically, patients with mild cases can improve their vision with glasses, while those with moderate to advanced keratoconus often need contact lenses. In severe cases, when contact lenses fail to offer satisfactory results, surgery becomes necessary [5].

Contact lenses help to smooth out corneal irregularities, providing a corrected and symmetrical anterior surface for the eye's refractive system at all stages of keratoconus [6]. Scleral contact lenses are rigid, gas-permeable lenses of large diameter that cover the entire cornea, forming a fluid layer situated between the back of the lens and the cornea. These lenses were first developed in the 19th century using glass and later polymethyl methacrylate, but their poor oxygen permeability restricted their use [7].

Scleral contact lenses, also referred to as scleral shells or haptic lenses, are categorized by the Scleral Lens Education Society based on their size. This classification includes semi-scleral lenses, which have diameters between 12.5 mm and 15 mm; mini-scleral lenses, which range from 15 mm to 18 mm; and large scleral lenses, which measure between 18 mm and 25 mm [8].

Surgical intervention, specifically corneal transplantation, also known as corneal grafting or keratoplasty, becomes a necessary treatment option for some patients with severe keratoconus. This approach is typically considered when nonsurgical management, such as specialized contact lenses, no longer provides adequate vision correction or when significant scarring has developed on the cornea. This procedure involves replacing the patient's damaged cornea with healthy corneal tissue from a donor [9].

Avoiding surgery in cases of severe keratoconus, as demonstrated in this case, presents significant benefits. Corneal transplantation, while often effective, is an invasive procedure that involves underlying risks, such as graft rejection, infection, high astigmatism, glaucoma, and prolonged recovery durations. By effectively managing severe keratoconus with scleral lenses, patients can preserve functional vision without undergoing these surgical risks and the associated psychological and financial burdens.

This case study aims to document the success of managing severe keratoconus with scleral contact lenses, thereby avoiding the need for surgery. This report will raise awareness among ophthalmologists and optometrists regarding the potential effectiveness of scleral lenses for severe keratoconus, subsequently allowing them to delay or prevent recommending keratoplasty.

## Case presentation

A 34-year-old man visited our contact lens clinic at Noor Alyemen Eye and E.N.T. Consulting Center, stating that he had experienced a significant loss of vision. He had been diagnosed with severe keratoconus in the past. His vision improved slightly with eyeglasses. Furthermore, he was evaluated and deemed ineligible for both corneal collagen cross-linking and intracorneal rings. The patient reported that multiple eye care providers had advised him to undergo corneal transplantation due to the severity of his condition. However, only one provider kindly referred him to our center for management with scleral contact lenses.

The patient stated that there was no family history of keratoconus and no prior episodes of vernal keratoconjunctivitis. His medical history was otherwise normal. Initially, the patient underwent standard eye examinations before the fitting process began. The exams checked the patient's vision, refraction, necessary eye dimensions, and the integrity of the anterior segment. Fundoscopy was also performed to evaluate the health of the posterior segment.

Uncorrected visual acuity was limited to counting fingers at 1 meter in each eye, with minimal improvement with glasses. The patient's best corrected visual acuity with spectacles for the right eye was 20/200 with a correction of -10.00/-4.00 x 60, and for the left eye, it was 20/400 with a correction of -12.00/-3.00 x 140.

A slit-lamp examination revealed typical signs of keratoconus in both eyes, in addition to a paracentral corneal scar in the left eye. All other aspects of the anterior segment were within normal limits. Fundoscopy showed a healthy posterior segment. The intraocular pressure, measured with a handheld tonometer (Icare Finland Oy, Vantaa, Finland) at 11:30 AM, was 8 mmHg for the right eye and 7 mmHg for the left eye.

Both eyes presented with identical anatomical measurements: a 12 mm horizontal visible iris diameter, an 11 mm vertical visible iris diameter, and a 10 mm palpebral aperture fissure height. The pupils were found to be 3 mm in diameter in bright light and 5 mm in diameter in dim light in both eyes. The tension of the eyelids was normal bilaterally, and the tear film was normal in both quantity and quality.

The four maps of Pentacam's corneal tomography were utilized to offer essential, detailed data for successful contact lens fitting. This comprehensive information, including precise corneal curvature and elevation details, primarily guides lens selection. Additionally, these maps are crucial for keratoconus staging and understanding cone morphology, both of which are vital for fitting specialized contact lenses.

**Figure 1:**
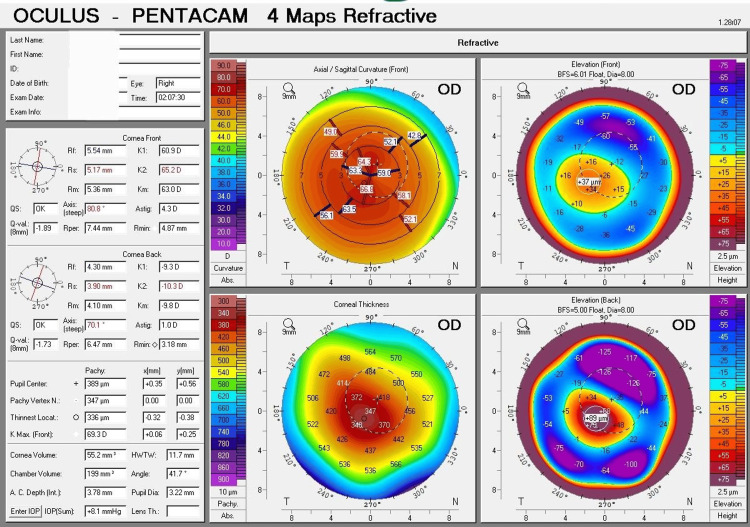
Refractive four maps of Pentacam corneal tomography (right eye) Refractive four maps of Pentacam corneal tomography for the right eye display severe ectatic changes that indicate the characteristics of severe (Grade 4) keratoconus. The curvature map exhibits marked steepening, with a Kmax of 69.3 D. The thickness map indicates an extremely thin cornea, with the thinnest location measuring 336 µm. The elevation map displays a significant protrusion, with an anterior elevation of 37 µm.

The essential measurements of the four maps can be summarized as follows: for the right eye, the corneal curvature displays a flatter curvature (K1) of 60.9 D (5.54 mm), a steeper curvature (K2) of 65.2 D (5.17 mm), an average curvature (Km) of 63.0 D (5.36 mm), a maximum curvature (Kmax) of 69.3 D, and an astigmatism of 4.3 D. The thinnest location of the cornea was 336 µm, while the thickness at the center of the pupil was 389 µm. The anterior surface's highest elevation was 37 µm, and the posterior surface's highest elevation was 89 µm (Figure 1).

**Figure 2:**
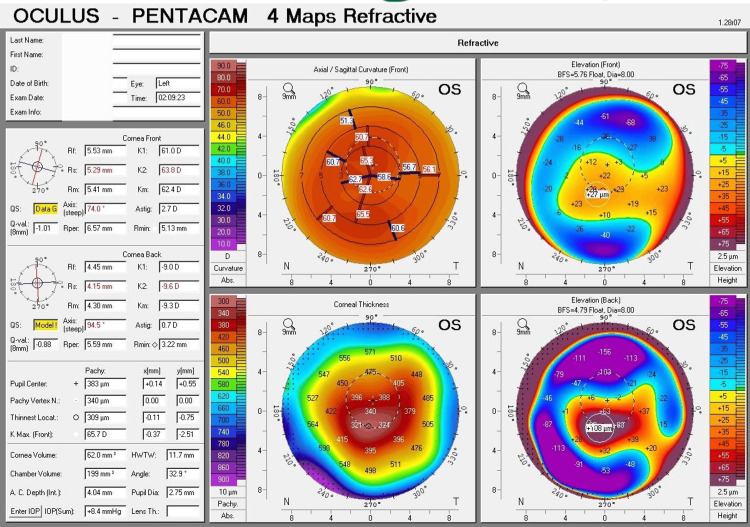
Refractive four maps of Pentacam corneal tomography (left eye) Refractive four maps of Pentacam corneal tomography for the left eye present abnormal ectasia indices indicative of severe (Grade 4) keratoconus. The curvature map shows pronounced steepening, with a Kmax of 65.7 D. The thickness map reveals an extremely thin cornea, with the thinnest location measuring 309 µm. The elevation map illustrates a significant protrusion, with an anterior elevation of 27 µm. In this image, the quality specification (QS) box appears yellow, indicating suboptimal scan quality due to the presence of a corneal scar.

The left eye measurements indicate a flatter curvature (K1) of 61.0 D (5.53 mm), a steeper curvature (K2) of 63.8 D (5.29 mm), an average curvature (Km) of 62.4 D (5.41 mm), a maximum curvature (Kmax) of 65.7 D, and an astigmatism of 2.7 D. The thinnest location of the cornea was 309 µm. At the same time, the thickness at the center of the pupil was 383 µm. The anterior surface's maximum elevation was 27 µm, and the posterior surface's maximum elevation was 108 µm (Figure 2).

After performing routine eye exams at our center, I determined that the patient is suitable for scleral contact lens wear, with no contraindications preventing their use. To properly fit the patient with scleral rigid gas permeable (RGP) contact lenses, I used a diagnostic trial lens method.

At the beginning of a scleral lens fitting, a fundamental initial step involves determining the total lens diameter. Our starting point for this crucial parameter is to take the horizontal visible iris diameter measurement and add 4 mm. This result provides a total lens diameter of 16 mm, which then serves as the baseline for selecting the first diagnostic scleral lens to be fitted to the eye.

Following the determination of the lens's total diameter (TD), the next essential step is to establish the sagittal depth. To guide this selection, I rely on the severity of the keratoconus, as indicated by corneal tomography data. Specifically, I utilize a starting point of 4400 µm for the initial lens sagittal depth. This value serves as a basic reference, which I will then refine based on the lens's on-eye performance and the resulting fluid layer observed during the fitting evaluation.

After identifying the initial trial lens parameters, I proceeded to clean, sterilize, and rinse the lens thoroughly. Next, I placed the lens onto the lens holder and filled it with normal saline. Then, I used a fluorescein strip to stain the saline solution. Following this, I carefully inserted the lens into the patient's eye. Subsequently, I instructed the patient to wait for an hour to allow the lens to settle optimally before I performed the fitting assessment.

Once the lens had settled for the recommended time, I began the fitting assessment. I started by checking the fluorescein pattern with the slit lamp, using diffuse illumination and cobalt blue light, which were within acceptable limits.

After assessing the fluorescein, I evaluated the clearance at the interface of the lens and the cornea. Using a slit lamp optic section set at a 45-degree angle, I measured this clearance at the highest point of the cornea and found it to be insufficient. Next, I evaluated the landing area and the interaction between the lens and the conjunctiva, finding this aspect acceptable.

**Figure 3:**
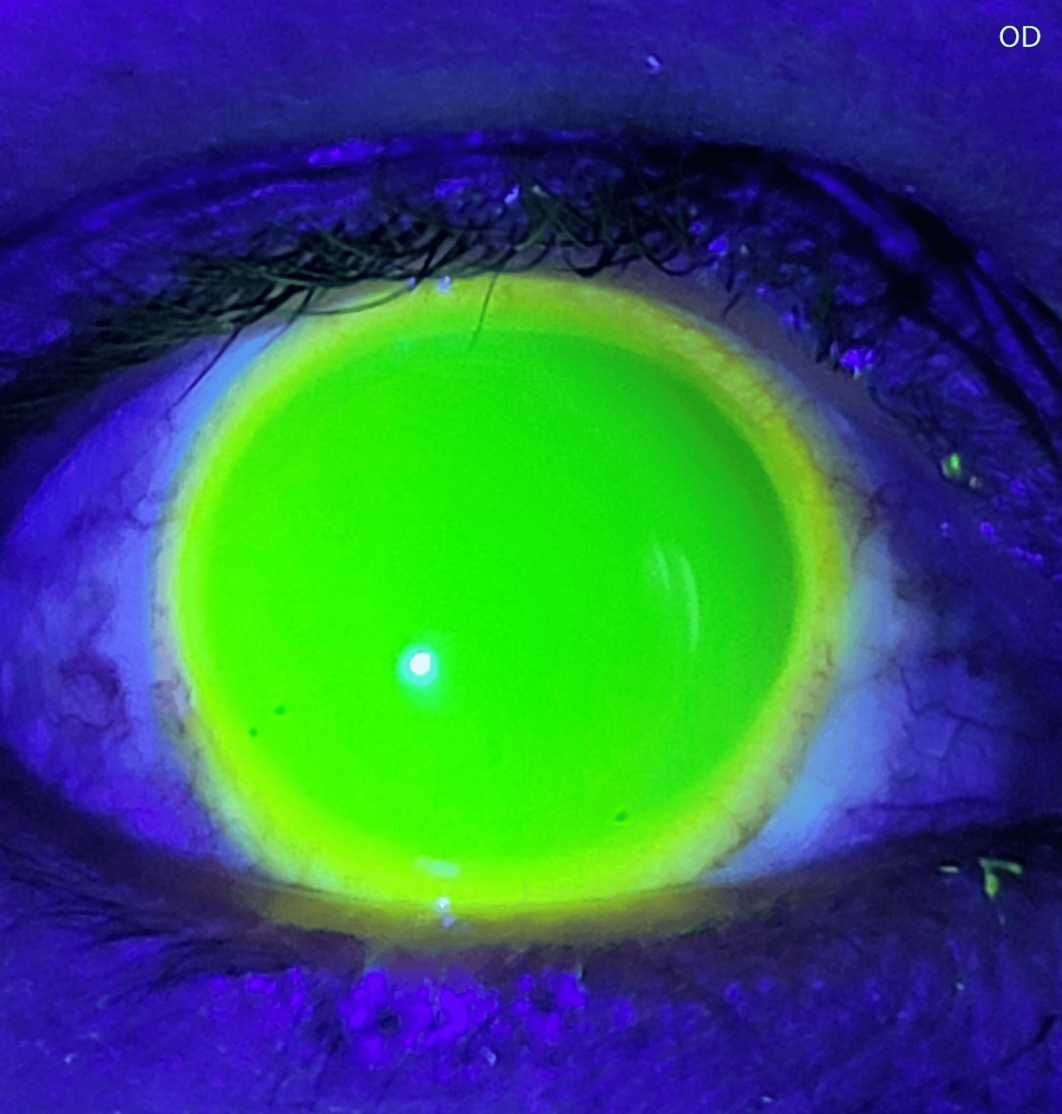
Fluorescein profile for the right eye The fluorescein-stained fluid reservoir under the scleral lens demonstrates a uniform dye distribution, indicating optimal corneal and limbal clearance, with no evidence of lens bearing on these sensitive structures.

**Figure 4:**
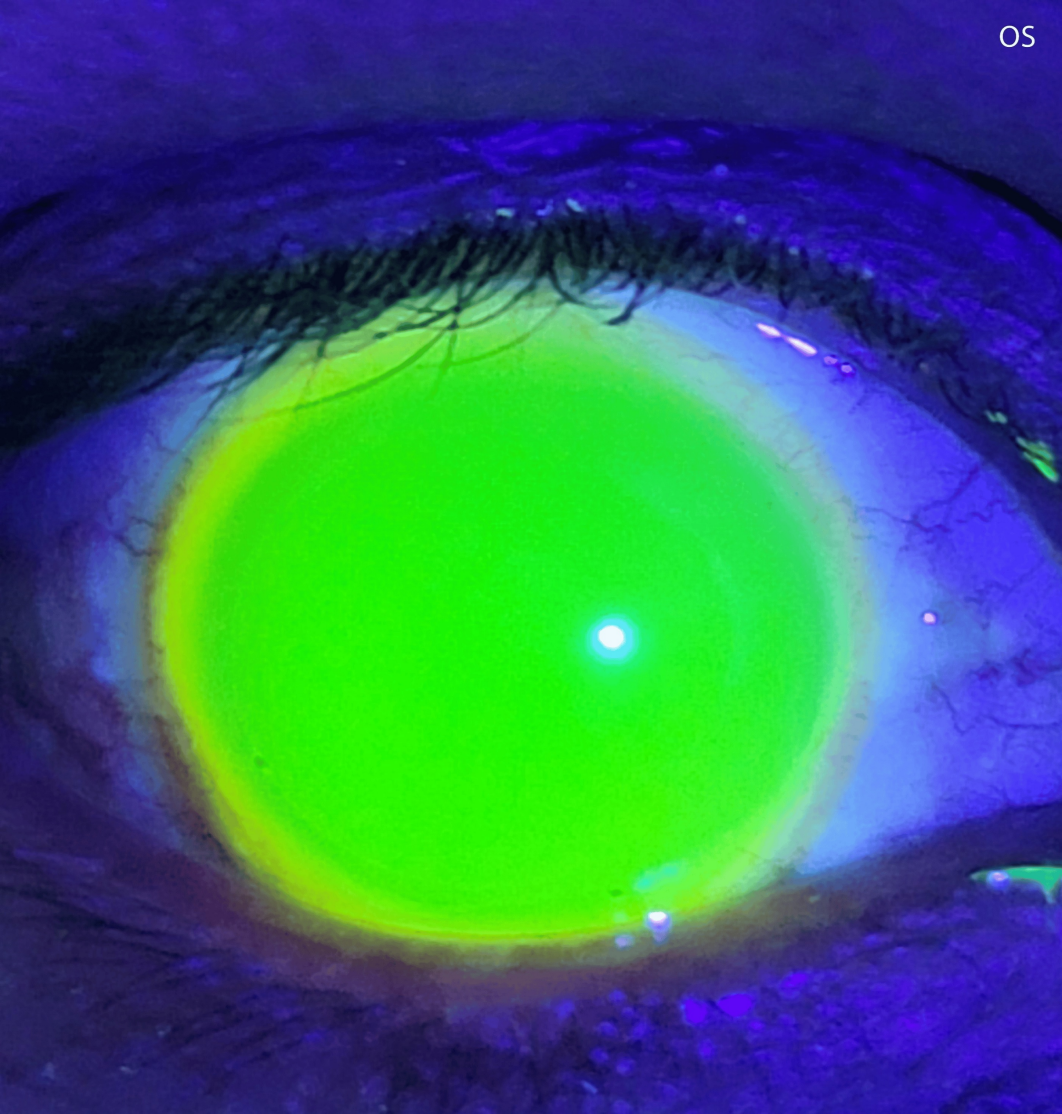
Fluorescein profile for the left eye The fluorescein-stained fluid reservoir beneath the scleral lens exhibits a consistent distribution of the dye, suggesting adequate corneal and limbal clearance and confirming that there is no lens bearing on these critical areas.

Due to the insufficient clearance, I exchanged the initial trial lens for one with greater sagittal depth. I subsequently repeated the previous evaluation process for each eye until I achieved an optimal fit. After that, I looked at the fluorescein profile again; I found it to be optimally distributed over the cornea and limbus (Figures 3-4).

**Figure 5:**
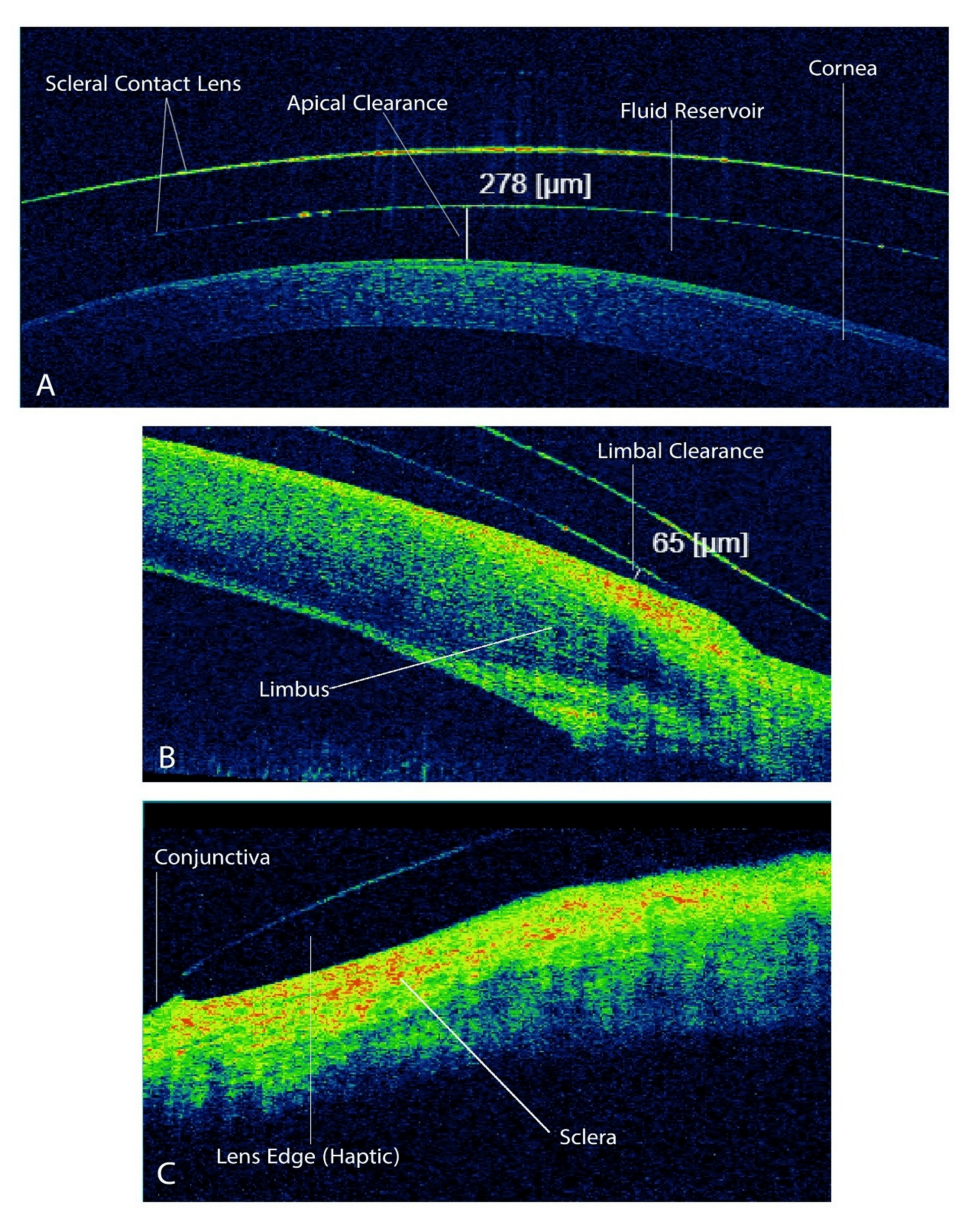
AS-OCT scan of the right eye The cross-sectional AS-OCT image illustrates the optimal scleral lens fit, showing the appropriate positioning of the scleral lens relative to the cornea and conjunctiva. (A) Reveals adequate apical corneal clearance of 278 µm; (B) shows sufficient limbal clearance of 65 µm; and (C) illustrates appropriate peripheral landing of the scleral lens (haptic) with no signs of excessive edge lift or edge impingement on the conjunctiva. AS-OCT: anterior segment optical coherence tomography

**Figure 6:**
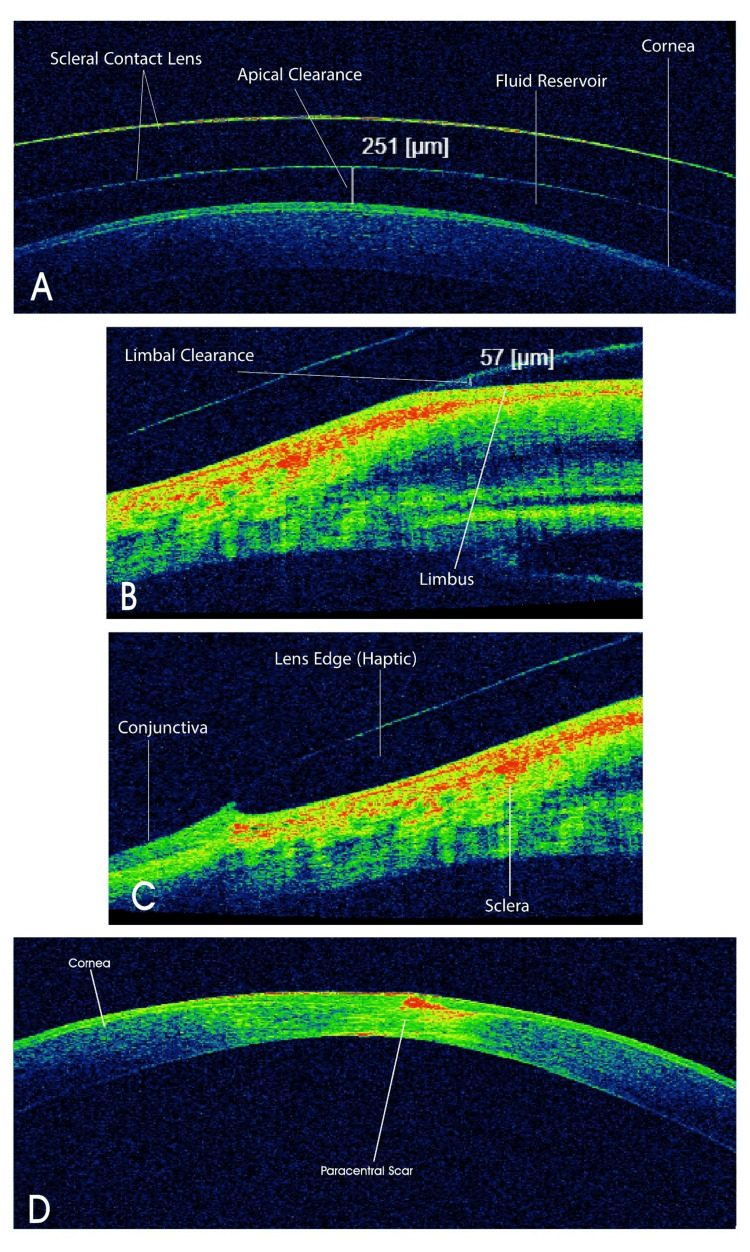
AS-OCT scan of the left eye The cross-sectional AS-OCT image demonstrates the optimal fit of a scleral lens. It highlights the proper alignment between the scleral lens and the cornea/conjunctiva. (A) Displays adequate apical corneal clearance of 251 µm; (B) exhibits sufficient limbal clearance of 57 µm; (C) visualizes smooth peripheral landing of the scleral lens edge (haptic) with no evidence of increased lens edge lift or conjunctival compression; and (D) illustrates the cornea without lens wear and demonstrates a paracentral corneal scar. AS-OCT: anterior segment optical coherence tomography

For optimal visual acuity, a careful over-refraction was conducted to establish the precise back vertex power (BVP) required for the prescribed lenses. During the scleral lens fitting evaluation for this patient, I used anterior segment optical coherence tomography (AS-OCT) to enable me to accurately measure the apical corneal clearance, limbal clearance, and the landing of the lens on the conjunctiva (Figures 5-6).

The contact lens fitting evaluation concluded with the identification of the patient's prescription parameters as follows: for the right eye, the TD is 16 mm, the BVP is -7.00 D, the base curve (BC) is 7.50 mm, and the sagittal depth is 4600 µm. For the left eye, the TD is 16 mm, the BVP is -9.00 D, the BC is 6.90 mm, and the sagittal depth is 4800 µm.

## Discussion

This report demonstrates the success of managing a severe keratoconus patient with scleral contact lenses, thereby avoiding the need for surgical intervention. The significant improvement in the patient's vision, from unaided visual acuity of counting fingers to achieving 20/30 with scleral contact lenses, emphasizes the powerful ability of this management approach to restore functional vision, even when keratoconus is severe.

Generally, keratoplasty is recommended for keratoconus patients when there is non-resolving corneal hydrops, corneal scarring, or when the best-corrected visual acuity does not improve beyond 20/40, and contact lenses are also intolerable [10].

Scleral lenses present clear advantages over corneal transplantation by eliminating surgical complications such as graft rejection, infection, prolonged healing, and high astigmatism. The fluid reservoir at the interface of the lens and cornea corrects irregular astigmatism while also mechanically supporting and hydrating the compromised cornea in severe keratoconus [5,9].

This patient was ineligible for corneal crosslinking or intrastromal rings due to extreme corneal thinning. In this case, conventional corneal RGP contact lenses were trialed but did not provide the desired results, so I decided to proceed with scleral contact lenses.

In this clinical scenario, Pentacam corneal tomography has been very helpful. Corneal tomography is a valuable tool for fitting scleral contact lenses because it offers a complete and precise picture of the cornea. Unlike keratometry, tomography illustrates the entire corneal surface, revealing irregularities and asymmetries that are crucial for fitting complex corneas, such as those with keratoconus or those that have been surgically operated on [11].

In this patient case, I also used AS-OCT. This device reveals precise details of the configuration between the scleral contact lens and the anterior segment of the eye. Through an accurate cross-sectional view, the OCT effectively illustrates the distance between the cornea and the lens (corneal clearance), the distance between the limbus and the lens (limbal clearance), and the lens edge profile, indicating whether the edges are appropriate or apply excessive pressure on the conjunctiva or stand off from it [12].

Bilgin et al. conducted a study that involved 518 patients with keratoconus, which revealed that the utilization of rigid gas-permeable contact lenses effectively postponed the necessity for surgery in 98.9% of the cases [13]. A study carried out by Zadnik et al. as part of the CLEK found that only 16% of keratoconus patients relied on glasses. In contrast, between 65% and 75% used contact lenses, particularly rigid gas-permeable contact lenses. For the 10% to 20% of cases that did not achieve satisfactory outcomes with these approaches, surgery was considered [14].

A study of 846 patients (1,692 eyes) who were followed at a cornea clinic for five years, conducted by Galvis et al., found that only 1.65% of patients (28 eyes) needed a corneal transplant, indicating that this surgery was not common among the clinic's patients during that time. Additionally, this study concluded that the use of scleral lenses reduces the need for corneal grafts in severe keratoconus, using the definition of Kmax ≥ 70 D [15].

Drawing from my clinical experience with keratoconus, many patients who have undergone keratoplasty still require scleral lenses to achieve good vision. This underscores the importance of scleral contact lenses as a key management tool for keratoconus, and prioritizing the use of scleral lenses can often help patients avoid surgery. Further evidence for my observations is found in numerous studies, such as a study by Severinsky et al., the conclusion of which is that scleral lenses are the best option for eyes with complicated corneal geometry because, in addition to providing visual rehabilitation, they may postpone or eliminate the need for additional surgery [16]. Moreover, Barnett et al. found that scleral lenses are safe, effective, and significantly improve vision in individuals who have undergone penetrating keratoplasty. Most patients report 20/40 vision or better [17]. Similarly, a study by Rocha et al. concluded that the use of mini-scleral lenses enabled effective visual improvement following keratoplasty, especially for patients who needed corrective lenses and were unable to use corneal RGP contact lenses [18].

During each follow-up visit with this patient, I consistently assess his level of satisfaction, which has remained high. The satisfaction experienced by scleral contact lens wearers frequently stems from the superior comfort and clear vision that these lenses provide, as well as the alleviation of dry eye symptoms [19].

This case study implies a few limitations. First, it was based on one patient, and the findings may not be applicable, since different patients with severe keratoconus may have different responses to scleral lenses. Second, due to the lack of a control group, it is not possible to state with certainty that scleral lenses are superior to other alternatives for any patient with severe keratoconus. Third, the absence of patient-reported outcome measures (PROMs) precludes a detailed investigation into patients' subjective experiences. Furthermore, a single follow-up visit does not provide a clear picture of how the condition has progressed and what other problems may develop in the future. Lastly, this report only mentions the positive aspects of scleral lenses while choosing not to discuss their drawbacks. However, compared to keratoplasty, these small issues of handling difficulty, midday fogging, and cost are negligible. This selective focus may restrict a balanced evaluation but reflects the clinical importance of prioritizing a non-surgical treatment in this case.

This study suggests that scleral contact lenses are the best option for managing severe keratoconus. It is also necessary to compare scleral lenses with other management approaches to determine which one works best for individual cases of severe keratoconus. Future studies should measure improvements in quality of life by using validated PROMs, such as the Ocular Surface Disease Index or the National Eye Institute Visual Function Questionnaire-25. It is important to continue monitoring patients for at least 12 to 24 months to confirm their treatment results and look for any late adverse effects.

## Conclusions

This case report practically proves the successful management of keratoconus, even in its severe stage, with scleral contact lenses, eliminating the necessity of keratoplasty. Despite the challenges presented by the condition and the patient's previous inability to achieve acceptable visual acuity with eyeglasses or other types of contact lenses, scleral lenses led to marked enhancements in vision and comfort. Using advanced instruments such as anterior segment OCT and Pentacam corneal tomography enabled me to attain the optimal scleral lens fit. The study results highlight that scleral lenses should be a first-line approach for managing severe keratoconus and may prevent the need for keratoplasty.
